# A Descriptive Review of Global Real World Evidence Efforts to Advance Drug Discovery and Clinical Development in Amyotrophic Lateral Sclerosis

**DOI:** 10.3389/fneur.2021.770001

**Published:** 2021-11-08

**Authors:** Suzanne F. Cook, Thomas Rhodes, Courtney Schlusser, Steve Han, Chao Chen, Neta Zach, Venkatesha Murthy, Shreya Davé

**Affiliations:** ^1^CERobs Consulting, LLC, Wrightsville Beach, NC, United States; ^2^Gillings School of Public Health, University of North Carolina-Chapel Hill, Chapel Hill, NC, United States; ^3^Takeda Development Center Americas, Inc., Cambridge, MA, United States

**Keywords:** amyotrophic lateral sclerosis, real-world data, rare disease, registries, biobanks

## Abstract

Understanding patient clinical progression is a key gateway to planning effective clinical trials and ultimately enabling bringing treatments to patients in need. In a rare disease like amyotrophic lateral sclerosis (ALS), studies of disease natural history critically depend on collaboration between clinical centers, regions, and countries to enable creation of platforms to allow patients, caregivers, clinicians, and researchers to come together and more fully understand the condition. Rare disease registries and collaborative platforms such as those developed in ALS collect real-world data (RWD) in standardized formats, including clinical and biological specimen data used to evaluate risk factors and natural history of disease, treatment patterns and clinical (ClinROs) and patient- reported outcomes (PROs) and validate novel endpoints. Importantly, these data support the development of new therapeutics by supporting the evaluation of feasibility and design of clinical trials and offer valuable information on real-world disease trajectory and outcomes outside of the clinical trial setting for comparative purposes. RWD may help to accelerate therapy development by identifying and validating outcome measures and disease subpopulations. RWD can also make potential contributions to the evaluation of the safety and effectiveness of new indications for approved products and to satisfy post-approval regulatory and market access requirements. There is a lack of amalgamated information on available registries, databases, and other sources of real-world data on ALS; thus, a global review of all available resources was warranted. This targeted review identifies and describes ALS registries, biobanks and collaborative research networks that are collecting and synthesizing RWD for the purposes of increasing patient awareness and advancing scientific knowledge with the hope of expediting future development of new therapies.

## Introduction

Amyotrophic Lateral Sclerosis (ALS) is a rare and fatal neurological condition with a prevalence of 6.8 cases per 100,000 people in the US, 5.7 per 100,000 people in the European Union (EU), 1.5 per 100,000 people in China, and 4.6 per 100,000 people in Japan (1). The personal, societal, and economic burdens of the disease are substantial (2).

Neurological conditions, including ALS, are particularly burdensome because many are chronic and lack curative therapies, occur throughout the lifespan, follow a progressive course, lead to relatively rapid functional impairments, and require significant health care resource and caregiver support (3). Within this context, ALS is associated with an especially aggressive course, typically leading to progressive paralysis and death within 2–5 years from symptom onset, thus leading to substantial unmet need to patients and their families.

ALS research, as in other rare disease research, benefits from collaboration between centers, regions and countries (4). Rare disease registries and collaborative platforms such as those developed in ALS collect real world data (RWD) in standardized formats. Clinical and biological specimen data are utilized to facilitate drug target discovery, evaluate risk factors and natural history of disease, treatment patterns and clinical and patient reported outcomes and validate novel endpoints. Registries can be useful in describing and characterizing medical therapies and performance measurement (5). Importantly, these data support the development of new therapeutics by supporting feasibility evaluations of clinical trials, providing much needed information about disease progression and sometimes also bring together patients to enable patient identification for clinical trial recruitment, all of which are challenging for rare diseases. They also offer valuable information on real-world disease trajectory and outcomes outside of the clinical trial setting for comparative purposes (4). Registry data is often highly generalizable to the source population and may be particularly useful in rare diseases where single center, observational studies and randomized controlled clinical trials are often infeasible (5).

RWD may help to accelerate therapy development by identifying and validating outcome measures and disease subpopulations. RWD can also make potential contributions to the evaluation of the safety and effectiveness of new indications for approved products and to satisfy post-approval requirements. Country and regional efforts have been developed in many parts of the world to help progress sustainable efforts at collecting real world evidence to accelerate therapy development in ALS.

The primary aim of this targeted review is to identify and describe ALS registries, biobanks and collaborative research networks that are collecting and synthesizing RWD for the purposes of advancing scientific knowledge and to inform the development of novel, safe and effective treatments and therapies through utilizing important patient data. Details of each registry are provided including strengths and limitations with respect to ALS research.

## Methods

A targeted literature search of the PubMed database was performed to identify papers that described RWD sources for ALS research. Population-based studies, national registries, biobanks, and disease-specific websites were included with a specific focus on the United States, Latin America, Europe, Japan, and China. Systematic reviews were included if published in 2015 or later. Additional targeted searches using Google and the reference lists from published articles supplemented the initial PubMed search.

The titles and abstracts of identified publications were initially screened, and full-text articles were examined to determine whether unique data sources were being described. Where available, the web sites of each identified registry or database were reviewed for detailed information on the nature of the data source.

Inclusion criteria comprised the following: (1) Publications describing RWD sources for ALS; (2) Published in the English language; (3) Published between January 2000 and March 2020; (4) Describes a source that could provide data that could be useful to support ALS research.

While the focus of the review was on larger scale collaborative registries, networks, and consortia, country-specific and regional registries and subsets were included if they were considered to be of potential value in supporting the development and delivery of RWD to advance ALS research and bring forward novel therapies to individuals with ALS. For completeness, subject matter experts were queried to provide input for potential data sources that were not identified in the literature search.

## Results

The identified registries and initiatives have various scientific objectives including quantifying the burden of illness in the population, describing and characterizing treatment patterns and outcomes, exploring and validating novel endpoints and the collection, storage and provision of biologic samples to the scientific community for the broad purposes of identifying novel opportunities for new therapies. Registry details, overall strengths and limitations, and recent papers using the identified registries are provided as available. Many of the efforts actively collaborate with organizations in areas of mutual interest with the common objective of accelerating therapy discovery and development and improving outcomes of patients with ALS.

### North America

Three population-based registries, two clinic-based registries, and one collaborative platform were identified in the US and Canada (see Table 1).

**Table 1:** Major population-and clinic-based ALS registries and collaborative research platforms in North America.

**Name**	**Sponsor/ Partner**	**Country**	**Enrolled patients**	**Data types**
**North American population-based registries**				
National ALS Registry^1^	Agency for Toxic Substances and Disease Registry (ATSDR). Centers for Disease Control and Prevention (CDC)	US	17,000 patients with confirmed ALS (2020)	• Sociodemographic characteristics, occupational history, military history, cigarette smoking, alcohol consumption, physical activity, family history of neurodegenerative diseases, and disease progression
National ALS Registry Biorepository	ATSDR, CDC	US	330 ALS patients with at least one biosample	• Biospecimens: blood, urine, hair, nail clippings; postmortem samples: brain, spinal cord, CSF, bone, muscle samples
Canadian Neuromuscular Disease Registry (CNDR)	University of Calgary	Canada	1,085 patients with ALS	• Clinical data, demographic characteristics, PROs • CRF available
**North American clinic-based registries**				
VA Biorepository Brain Bank	Veterans Affairs (VA)	US	287 veterans with ALS	• Clinical data, demographic characteristics, PROs; neurologic tissue specimens
Muscular Dystrophy Association (MDA) ALS Registry/MOVR Data Hub MOVR: NeuroMuscular ObserVational Research	MDA	US	2,685 patients at MDA clinics across country	• Comprehensive clinical, PRO, genetic data; pts seen at MDA clinics • Incorporates data from smartphones, wearable devices
**North American consortia/collaborative platforms**				
Target ALS	Target ALS	US		• Makes results broadly available to ALS researchers • Makes available tissue specimen and samples, associated clinical data
NEALS (Northeast Amyotrophic Lateral Sclerosis Consortium)	NEALS	US	342 patients, including 28 cases of ALS, 163 cases of non-neurologic controls, and 151 cases of neurologic controls (non-ALS)	• Makes results broadly available to ALS researchers • Makes available tissue specimen and samples, associated clinical data
CReATe Consortium (Clinical Research in ALS and Related Disorders for Therapeutic Development)	CReATe	US	750 patients including sporadic, familial and presymptomatic mutation carriers	• Makes results broadly available to ALS researchers • Makes available tissue specimen and samples, associated clinical data
Genomic Translation for ALS Care (GTAC)	GTAC	US	1,500 patients	• Clinical data, demographic characteristics, PROs; neurologic tissue specimens and samples
Answer ALS	Answer ALS	WW	1,000 patients from 10 centers	• Clinical data, demographic characteristics, PROs; neurologic tissue specimens and samples

#### North American Population-Based Registries

##### The US National ALS Registry

The US National ALS Registry is the national surveillance system for ALS and is the largest research project designed to identify cases from the entire US population. Every person with ALS in the US is eligible to enroll in the National ALS Registry^2^.

The registry uses existing administrative data as a primary source of case ascertainment as well as self-enrollment. The Agency for Toxic Substances and Disease Registry (ATSDR) launched the National Registry using a two-step enrollment approach. The first step used existing national administrative databases, including Medicare, Medicaid, Veterans Health Administration, and Veterans Benefit Administration records to identify prevalent cases of ALS. The second step utilized a secure web portal to capture cases not already included in the administrative databases. There were approximately 17,000 patients with ALS in the registry. Patients ranged from ages 18–80+ years and were majority male (60%). Patients were overwhelmingly of white race (91%), and lived in all major census regions (Midwest, South, West, East). The majority of patients reported initial site of onset as the limb (72%) vs. bulbar (22%) or trunk (6%) (6).

ALS registrants complete a detailed case report form (CRF) and risk factor surveys including sociodemographic characteristics, occupational history, military history, cigarette smoking, alcohol consumption, and physical activity. The registry also collects family history of neurodegenerative diseases and disease progression, and administers patient surveys on residential history, pesticide exposures, occupations and hobbies involving toxic exposures, trauma, dietary patterns, reproductive history, and future reproductive plans.

The ALS National Registry sponsors extramural research in several key areas of ALS etiology, risk factors and exploratory endpoints. Recently funded extramural research includes numerous studies of environmental (e.g., persistent organic pollutants, ambient air pollution) exposures, other risk factors (e.g., antecedent medical conditions and medications), environment and genetic interactions, novel biomarkers and ALS natural history and progression.

The registry references and collaborates with state-wide registry efforts and other state-based Comprehensive Surveillance Projects. A study on environmental risk factors and gene-environment interaction references collaboration with Clinical Research in ALS for Therapeutic Development (CReATE) which is part of the Rare Diseases Clinical Research Network (RDCRN).

Kaye et al. (7) assessed the completeness of the National ALS registry by comparing persons with ALS who were passively identified by the registry with those who were actively identified in the State and Metropolitan Area ALS Surveillance Project (SM ALS). The SM ALS project was created to support the development of the National ALS Registry and to evaluate completeness of the registry as it developed using active case ascertainment (physician reporting, medical records abstraction, and death certificates in several states and metropolitan areas selected in part to over-represent racial and ethnic minorities.

Using the SM ALS, Kaye et al. (7) identified 5,883 cases of ALS; of those cases, 1,116 died before the National ALS Registry was initiated. Of the remaining 4,767 cases eligible, 2,720 (57%) matched a case that was identified by the Registry. Cases identified by the SM ALS Project that did not match cases in the registry were more likely to be non-white, Hispanic, <65 years of age, and from Western states. There were significant differences in the stage of ALS at the time of the report and types of insurance used; those who were diagnosed with possible or unclassified ALS and those who did not use Medicare were less likely to be identified by the registry (7). The authors concluded that the methods used by the registry to identify cases of ALS worked relatively well, however, the registry missed cases, suggesting that developing strategies to identify and promote the registry to those who were more likely to be missing could be beneficial to improving the completeness of the registry (7).

Since 2014, the National ALS Registry data have been used to publish over a dozen studies on topics including the etiology, genomics, risk factors, and correlation of environmental exposures with ALS [e.g., (8, 9)].

##### National ALS Registry Biorepository

A biorepository, comprised of biospecimens from persons with ALS enrolled in the National ALS Registry was created in 2011. The initial research project comprised a pilot study of two data collection efforts: blood, urine, hair and nail clippings from 300 ALS patients and postmortem specimens in up to 30 deceased patients. An additional feasibility assessment of the development of fibroblast cell lines from post-mortem cell lines was also initiated.

The pilot study demonstrated that a nationwide collection of pre- and post-mortem biospecimens from the National ALS Registry enrollees is feasible, warranted, and can be done in a cost-effective manner (10). Currently, in-home samples from 1,411participants to 48 post-mortem individuals are included in the biorepository^3^.

The biorepository is a subset of the National Registry and has promise for the exploration of natural history and endpoint data in ALS; and the findings of feasibility and cost effectiveness from the pilot study were compelling. The ability to link the registry data with the biorepository data is noteworthy and may make the biorepository data a valuable resource in clinical development. The linkage between the datasets is a significant opportunity for observational research and advancement of novel therapeutics. Researchers who wish to analyze data from the biorepository can obtain linked epidemiological data from the National ALS Registry, including military history, family history, and occupational history. Samples are accessible to researchers across the world, regardless of institutional affiliation. There is a cost associated with obtaining samples from the biorepository.

##### Canadian Neuromuscular Disease Registry

The Canadian Neuromuscular Disease Registry (CNDR) is a population-based registry established in 2011 to connect adult and pediatric Canadians living with a neuromuscular disease with national and international research opportunities^4^. The CNDR collects data from patients on over 140 different neuromuscular diseases, including ALS, across 10 academic institutions and 28 clinics, including 10 multidisciplinary ALS clinics. The majority of participants enrolled in the registry are enrolled during routine clinic visits by a research coordinator or clinician. There is also the option to self-register by contacting the CNDR National Office. The CNDR operates as a network of university-affiliated neuromuscular clinics and local principal investigators.

As of 2018, there were 1,085 ALS patients enrolled in the CNDR with complete data (11). The mean age at onset within the population is 60 years; the mean age at diagnosis within the registry is 62 years. The population is majority male (60.3%), and the median survival within the registry is 36.5 months. Data included in the ALS registry include diagnosis [The El Escorial criteria (EEC), family history, symptom onset], clinical features (functional data, pulmonary function test, cognitive impairment and behavioral impairment), and social data (transportation, home care, lifestyle information, occupation, income, military status, and education). Data is available to any interested parties following a brief review process.

Hodgkinson et al. (11) assessed provincial differences in the diagnosis and care of ALS in the CNDR. Of the 1,085 patients with available data, the authors analyzed 1,006 patients, excluding 79 patients with incomplete data. Using international prevalence data and Canadian population figures, the authors estimated that there were approximately 2,800 living ALS patients in Canada. Findings from this study indicated that the mean time of symptom diagnosis differed significantly across provinces (*p* = 0.003). There was no significant difference among time to diagnosis across provinces for males compared to females (*p* = 0.255). Riluzole usage was also significantly different across provinces (*p* < 0.0001).

#### North American Clinic Based Registries

##### VA Biorepository Brain Bank (VABBB)

The Veterans Affairs Biorepository Brain Bank (VABBB) is a national prospective cohort study and human tissue bank that collects, processes, stores, and provides research specimens for future scientific studies focused on neurological diseases that impact US Veterans. The VABBB conducts biannual follow-up telephone interviews until the veteran's death. Veterans without neurological diseases are also eligible to participate in the VABBB to serve as healthy controls.

The VABBB provides central nervous system tissue and health information to scientists studying ALS, disorders of veterans of the 1990–91 Gulf War, post-traumatic stress disorder, and other neurological conditions. It has enrolled 287 veterans with ALS since its inception in 2006. In 2011, active recruitment of veterans with ALS began via VA neurology clinic referrals, through word of mouth, or the VABBB website. All referrals meet EEC for possible, probable, or definite ALS as determined by the referring neurologist.

Over the past 10 years, the VA has assembled a large inventory of whole brain and spinal cord samples from Veterans with ALS and control participants. Veterans from across the U.S. are enrolled in the study and enrollment is ongoing. Regular follow-up calls with participants and their families collect functional, cognitive, health, and demographic data. A diagnostic neuropathology report is completed for all participants postmortem. Most samples undergo analysis for known ALS genetic mutations.

The VABBB began collaborating with the National Disease Research Interchange (NDRI), a human tissue research center, to assist in tissue recoveries in 2014; this collaboration has streamlined the tissue recovery process based on publicly available data. Researchers may submit proposals to the VABBB for review and provision of samples.

Brady et al. (12) described participants enrolled in the VABBB. Among evaluable patients at the time of the study (*n* = 133), the current mean age was 68.3 years; among those deceased (*n* = 100), the mean age at death was 68.0 years. The population of participants were majority male (96% living, 93% deceased), and white, non-Hispanic (97% living, 99% deceased). Additionally, the majority of participants experienced sporadic ALS (97% living, 94% deceased).

Researchers have recently begun studying chronic traumatic encephalopathy in ALS patients using the VABBB data. Walt et al. (13) analyzed 287 VABBB participants with a diagnosis of ALS. Of those participants, 156 were deceased with complete neuropathological evaluations. The ability to study these patients may represent a significant opportunity for research in risk factors and natural history of the disease.

Registrants in this specialized, clinic-based registry are a unique patient population of those veterans who are treated at VA clinics and data do not necessarily reflect what would be collected in the population of US Veterans as a whole. A compelling strength of this database is the ability to link VA medical records data with the tissue data.

##### Muscular Dystrophy Association: U.S. Neuromuscular Disease Registry

The Muscular Dystrophy Association (MDA) initiated the US Neuromuscular Disease Registry for seven diseases, including ALS, in 2013. The registry is comprised of ALS patients who receive care at 26 MDA Care Centers across the US. MDA is a care-based registry focused on treatment patterns and outcomes.

The overarching aim of the MDA registry is to optimize the clinical care of patients in order to improve survival and quality of life and to advance drug development. The specific goals of the MDA registry are to gain a better understanding of the natural history of illness, collect genotype-phenotype information to allow for better prediction of disease natural history, identify best clinical practices by collecting longitudinal data, utilize registry data as a platform to implement quality improvement in care, generate outcome-related information for patients and their families, establish a database of individuals to assist with clinical trial recruitment and accelerate drug development.

There are approximately 1,500 ALS patients currently enrolled in the MDA Registry, including more males than females. A majority of the participants are in the age range of 50–69 years (median age at reported symptom onset: 59 years; median age at diagnosis: 61 years). The majority of individuals with ALS in the registry received an ALS diagnosis within the last 2–5 years (14).

Data collected includes demographics, diagnostic test results, standardized measures of muscle function and health status, other clinical metrics, and records of medical interventions. The registry matches medical data with individual's genetic information where available, and data are updated during every MDA Care Center visit, providing continuity, and enabling longitudinal recording of symptoms.

It is reasonably hypothesized there are regional differences, and perhaps differences related to resources or economic variables that should be explored and quantified. No published papers on the ALS Registry were identified, though the registry is fairly recent in inception.

#### North American Consortia and Other Collaborative Platforms

##### Target ALS

Target ALS, a US national foundation founded in 2013 is an organization committed to bringing together ALS research, patient samples and preclinical tools through an Innovation Ecosystem model, fostering unprecedented scientific collaboration between academia and the pharmaceutical/biotech industry. This includes, among others, enabling access to a range of critical tools ranging from biospecimens to genomic datasets to stem cells through core centers for postmortem tissue (15), genetic, transcriptomics and proteomics data (16), as well as access to patient cells. Making these data and samples available for the community had been associated since 2013 with over 100 publications and enabled target identification and validation, with some key targets such as risk factor ATXN2 already reaching clinical trials (17).

##### NEALS

NEALS (Northeast Amyotrophic Lateral Sclerosis Consortium) was founded in 1995 with the goals of enabling both research tools and clinical trial network, with nine academic clinical centers in the New England region of the US and since then expanded significantly. NEALS is funded primarily by the ALS Association's TREAT ALS Network, Muscular Dystrophy Association, and various donors, and has grown to 140 research centers performing research related to ALS. The majority of these centers are US-based, but an increasing number of member sites are based internationally. NEALS is comprised of member sites, which are medical institutions that are equipped to perform clinical trials in ALS.

NEALS maintains and makes available to outsider researchers the de-identified data from clinical trials, and currently maintains five ALS trial databases. The trial databases available for researchers include: (1) a randomized, placebo-controlled trial of topiramate in amyotrophic lateral sclerosis, (2) trial of celecoxib in amyotrophic lateral sclerosis, (3) a clinical trial of creatine in ALS, (4) tolerance of high-dose coenzyme Q10 in ALS, and (5) study to investigate the safety and efficacy of lithium in volunteers with amyotrophic lateral sclerosis. In addition to the trial databases that NEALS maintains, researchers are invited to share their work on the NEALS site to make the trial results available to patients and potential subjects.

Additionally, NEALS and the Massachusetts General Hospital have created a repository of serum, plasma, cerebral spinal fluid, whole blood, extracted DNA, and urine samples from NEALS research studies of ALS. The sample repository is partially funded by the ALS Association, and samples are available to researchers to further understand ALS and its developing disease biomarkers. While NEALS is not a registry of ALS patients, this initiative is included because of its collaborative effort of 140 medical institutions in the US to advance ALS research and share results with the scientific community.

##### CReATe

CReATe (Clinical Research in ALS and Related Disorders for Therapeutic Development) is funded primarily by the National Center for Advancing Translational Sciences (NCATS) and the National Institute for Neurological Diseases and Stroke (NINDS) as part of the NIH Rare Diseases Clinical Diseases Research Network, with additional support from the ALS Association and Target ALS to enhance the CReATe Biorepository. Similar to Target ALS and NEALS, while this initiative is not a registry effort *per se*, it is included because of the breadth of the collaborative effort, the primary scientific objective to advance and validate biomarker candidates, and the intention to share results with the scientific community.

CReATe will take a series of biofluid biomarker candidates and provide the validation necessary to implement them in upcoming clinical trials. The patient samples and clinical information are collected by CReATe Consortium investigators at 15 academic institutions. The candidates include urinary p75 neurotrophin receptor extracellular domain (p75ECD); blood and cerebrospinal fluid (CSF) phosphorylated neurofilament heavy (pNfH); and blood and CSF neurofilament light (NfL). Data within the biorepository include biological samples of DNA, plasma, buffy coat (the blood component containing leukocytes and platelets), serum, RNA, peripheral blood mononuclear cells (PBMC), CSF, and urine.

The intended research objectives and projects are promising particularly in the area of innovative biomarkers and endpoints, and the collaboration with the MDA and numerous pharmaceutical companies is notable. For example. CReATe consortium expanded a previous initiative, pre fALS, targeting carrier of ALS familial mutations, which enabled the launch of a prevention study in ALS using disease related biomarker NfL in the inclusion criteria Additional information on the participating institutions and patient recruitment and enrollment in the CReATe consortium would be informative to assist in further assessing the effort (18).

As an example, for the collaborative model of both CReATe and Target ALS, recently Target ALS announced the launch of an initiative that connects the CReATe Consortium, the MDA, and 10 pharmaceutical companies to validate the potential ALS biomarker candidates, and make results broadly available to ALS researchers.

##### Genomic Translation for ALS Care (GTAC)

A collaboration between Columbia University, the ALS association and Biogen, this initiative aims to set the stage for a nationwide effort to ensure the genomic characterization of all people with ALS. The study will follow 1,500 people living with ALS in the clinic over a 3 year period with 3 month visit intervals to collect clinical data, sequence their DNA and store blood samples to generate induced pluripotent stem cells (iPSCs). This information will allow the correlation of ALS clinical manifestations to the genetic causes and help stratify patients for future clinical trials. For example, jointly with CReATe and Answer ALS, the consortium recently enabled the identification of KIF5A as a novel ALS Gene. Ultimately, the initiative will provide a basis for the development of precision medicine, or more individually tailored therapies for ALS (19).

##### Answer ALS

Answer ALS is a global project dedicated to developing and implementing a unified strategy to stop Amyotrophic Lateral Sclerosis (ALS). It originated as a result of the 2013 ALS Team Gleason Summit, which brought together leading researchers, patients, caregivers and advocates. The event was spearheaded by former NFL player Steve Gleason who lives with ALS and founded the ALS advocacy group, Team Gleason and was funded by Jay Fishman, former Chairman and CEO of The Travelers Companies, ALS Finding a Cure (a project of the Leandro P. Rizzuto Foundation), the National Football League, the PGA TOUR, The Bari Lipp Foundation, American Airlines, Warlick's Warriors' Aviators Against ALS, Stay Strong vs. ALS, and Microsoft. The initiative includes six US clinics, collecting comprehensive clinical data, biological samples [whole blood, plasma, serum and, optionally, cerebrospinal fluid (CSF)], patient cells, and comprehensive DNA, RNA and proteomics datasets. Data from all arms of the program are stored, integrated and analyzed on a secure cloud server. The data enable target identification and validation, as well as development and validation of novel endpoints (20).

### EU

In Europe, there is a long-standing ALS registry collaborative effort that largely arose from successful country and/or regional ALS registries initiated in the late 1990's. Individual country registries may have additional research objectives, but the European collaborative generally seeks to quantify the burden of illness and clinical features of ALS in the population.

#### EU Population-Based Registries

##### EURALS

Following the successful development and execution of country-based registries in countries including Scotland, England, Ireland and selected regions in Italy, the European ALS Consortium (EURALS) was established by the European Neuromuscular Center (ENMC) (2019).

The mission of the ENMC is to encourage and facilitate communication and collaboration in the field of neuromuscular research. The aim is to improve diagnosis and prognosis, find effective treatments and optimize standards of care to improve the quality of life of people affected by neuromuscular disorders. Researchers and clinicians from numerous countries are included in the collaborative, and the ENMC implemented a database for patients with ALS from participating countries and provides updates on epidemiology of both sporadic and familial ALS.

The data contributed to a case-control study of 575 people with ALS and 1,150 matched controls in Italy, Ireland, France, UK, and Serbia. The population-based study identified traumatic events that led to functional disability or that were confined to the head as risk factors for ALS, particularly in people aged 35–54 at the time of the event (21).

##### ENCALS

The European Network to Cure ALS (ENCALS) is a network of ALS centers and regional registries across Europe. ALS Center membership is open to all universities and hospitals in Europe with ALS clinical/research activity.

The broad aims of ENCALS are a recognized, visible, active and efficient collaboration effort, harmonized collection and storage of data on a European level, attraction of pharmaceutical companies to conduct ALS trials in Europe, and facilitation of patient-oriented research projects. Researchers and clinicians within the network collaborate on clinical trials, validation of diagnostic markers, treatment guidelines and scientific publications, uncovering disease mechanisms and translating basic research into treatment development (22). The network also provides broad EU-wide expert opinion and position statements in ALS, as they did in the case of Edaravone, who was considered for regulatory approval in the EU (23).

ENCALS seeks to initiate collaboration between funding agencies, reach consensus on research projects, and study novel designs for the assessment of the efficacy of new pharmaceuticals in ALS. The broad registry effort aims to be population-based and includes a combined total base population of approximately 24 million people and a combined total of 1,028 ALS cases. Within each European register, approximately 60–100 ALS cases are collected each year.

The ENCALS uses standardized data collection instruments and the same EEC diagnostic criteria across countries.

Data collection is comprehensive and includes demographic, clinical, disease trajectory, outcome and genetic data. Data on cognitive, behavioral and psychiatric co-morbidities are collected, and numerous genetic tests are conducted. Although ALS is a disease that affects the patient physically, it also has an impact on a patient's mental health; as such, these data may be important to the patient and caregiver.

Logroscino and Piccininni (24) assessed the descriptive epidemiology within the ENCALS data with the objective of evaluating geographic differences. ALS incident cases were drawn from a source population comprising almost 24 million people across Europe (ALS cases: 1,028). The incidence was 2.2 per 100,000 person years for the general population. In contrast, it was noted that other population-based studies have measured the lowest incidence in East Asia to be 0.89 per 100,000 person years and in South Asia to be 0.79 per 100,000 person years. The authors report that the origin of geographic difference in ALS incidence is a matter of debate, possibly due to genes and environmental risk factors.

##### TRICALS

A third and newer EU-based patient data collaboration is Treatment Research Initiative to Cure ALS (TRICALS) (25). Aims to (1) unify academia, patient advocacy groups, industry partners and funding bodies toward a common goal in finding a cure for patients, (2) provide a harmonized, international infrastructure for the conduct of clinical trials and (3) coordinate research efforts that maximize the likelihood of successful drug development. Currently, the TRICALS consortium consists of 40 specialized centers in 14 countries, diagnosing over 3800 patients with ALS each year.

#### EU Country Specific/Regional Registries

Several local or regional registries from the EU were identified, and a sample are summarized below in Table 2. These registries are generally population-based as noted, and data collected are generally similar to that collected by the ENCALS registry (24, 26–33).

**Table 2:** Regional registries: selected list.

**Region**	**Year initiated**	**Objective**	**ALS cases (*N*)**
Swabia, Germany (26)	2008	Incidence, patient and disease characteristics, natural history of ALS	438 (2014)
Ireland (The Irish ALS Register) (27)	1994	Incidence, prevalence, clinical features; associated DNA bank	2,128 (1995–2017)
Southeast England ALS Register (SEALS) (28)	2002	Incidence, patient and disease characteristics, natural history of ALS	138 (2002–2006)
Puglia, Italy (Sclerosi Laterale Amiotrofica- Puglia, SLAP) (29)	1997	Incidence, prevalence, disease characteristics of ALS	130 (1998–1999)
Piedmont and Aosta Valley, Italy (PARALS) (30)	1995	Incidence, prevalence, disease characteristics of ALS	2,702 (2014)
Lombardy, Italy (31)	1998	Incidence, prevalence, disease characteristics of ALS	517 (1998–2002)
Scotland (Clinical Audit Research and Evaluation of MND CARE-MND) (32)	1989	Clinical and epidemiological features of MND. Prospectively identify incident patients	406 (2015–2016 alone)
Spain (Sant Pau-MND (SP-MND)) (33)	2004	Incidence, prevalence, disease characteristics of ALS	294 (2004–2015)
European Union, EU Wide Initiative (EURALS, ENCALS, TRICALS)	1990	Incidence, prevalence, disease characteristics of ALS	5,403 (2019)

The identified registries generally appear to collaborate with broader registry efforts in Europe and generally use the same data collection form as the ENCALS referenced above.

The long-standing nature and sustainability of some of the databases is notable with some registries initiated over 30 years ago. As such, these registries may be sources for important historical data to inform clinical development.

#### EU-Based Transnational Collaboration

Starting out in the Netherlands and expanding internationally, Project MinE^5^ plans to map the full DNA profiles of at least 15,000 people with ALS and 7,500 healthy volunteers to conduct comparative genetic research with the aim of identifying the genetic cause of ALS to inform treatment approaches. The project was initiated by patients with ALS and is a worldwide collaboration between ALS centers and foundations. The project web site includes a variant browser based on over 6,400 whole genomes and burden test results for over 15,000 genes^6^. Data from the variant browser contributed to a recent publication which helped to identify KIF5A as a novel ALS gene (19).

### Other Parts of the World

#### Latin American and South America

The ENCALS consortium model has been extended to three Latin and South American countries: Cuba, Chile, and Uruguay to form the Latin American Epidemiology Network for ALS (LAENALS). Recruitment of patients for the LAENALS is ongoing; the data will be combined with the ENCALS data to build capacity and facilitate statistical analysis of risk factor and natural history data in ALS.

The objective of LAENALS is to execute comparative population-based ALS research in 3 Latin and South American countries characterized by different levels of genetic cross-linking, and to compare incidence, demographics, and phenotype with population-based data from European countries collected as part of the EURALS Consortium.

LAENALS will evaluate diverse ethnicities in the same territory to evaluate the possible role of genetics, socioeconomic status and other demographic characteristics of patients with ALS. Patients in this registry complete the same CRF as the European consortium registries. This registry effort is in its beginning stages and no published papers were identified. However, LAENALS have reported preliminary data on their website.

In Cuba, using mortality data from 432 ALS deaths, the adjusted mortality rate for the population over 15 years old was calculated as 0.83 per 100,000 person-years (PY). Adjusted mortality rates were significantly lower in the mixed population (0.55/100,000 PY) than in whites (0.93/100,000 PY) and blacks (0.87/100,000 PY). Over 100 new cases of ALS were prospectively identified into the registry for further follow up (34).

In Chile, a preliminary study on mortality has been completed. The authors used data from 1,671 deaths registered during 1994–2010 to calculate the crude and adjusted mortality rate of the Chilean population. The last 5 year analysis showed a crude mortality rate of 1.13 per 100,000 inhabitants (35).

A more recent study used LAENALS data to compare the clinical and genetic features of ALS across Cuba and Uruguay with Ireland. While no differences in survival were observed, Cuban and Uruguayan patients had a younger mean age of onset and Cuban patients were more likely to carry the C9orf72 repeat expansion compared to Irish patients (36).

While this is a relatively new effort, it appears to be the first large-scale, population-based registry initiative in these countries. The alignment to the large-scale and experienced European collaborative is notable. As such, the initiative has promise for informative epidemiologic and clinical data in regions that may be under-represented in population-based research on ALS. The particular focus on genetic data in this initiative and the plan for comparative analysis with the European registry data is notable.

#### Japan and China

Sources of registry data from patients with ALS in Asia are limited. One ALS research consortium in Japan and one hospital-based registry in China were identified (Table 3).

**Table 3:** ALS registries and collaborative research platforms in Latin America, South America, Japan, and China.

**Region/Country**	**Year initiated**	**Objective**	**ALS cases**
Latin/South America: Cuba, Chile, and Uruguay^7^	2016	To carry out comparative population studies and a case-control study of environmental risk factors for ALS across 3 Latin American countries	Not available
Japanese Consortium for ALS research (JaCALS) (37)	2006	To investigate the longitudinal course of Japanese patients with ALS	1245 (2006–2017)
West China Hospital of Sichuan, (38)	2006	Unknown, but publications have aimed to profile clinical features of ALS in southwest China	1201 (2006–2014)

##### Japanese Consortium for ALS research (JaCALS)

Japan maintains a multicenter registration and follow up system called the Japanese Consortium for ALS research (JaCALS) launched in 2006. No English language papers were identified; available data from abstracts are presented below.

JaCALS was initiated to investigate the longitudinal course of Japanese patients with ALS. Data from the JaCALS describes and characterizes the natural history of ALS including genetic background and the clinical and genetic factors associated with disease progression and prognosis. As of March 2021, 33 neurology institutions were participating in the JaCALS with over 900 patients with ALS registered (39).

Genomic DNA samples and B-cell lines are stored and linked with clinical data. The utility of this dataset is difficult to assess due to the limited availability of information; consultation with Japanese investigators is recommended to gain further data and allow for assessment.

##### West China Hospital of Sichuan, China

China does not appear to have a national, population-based ALS registry based on a review of publicly available information. The Department of Neurology of the West China Hospital of Sichuan University, Chengdu, Sichuan province, established a hospital-based register of all cases of MND in 2006 to the present. This hospital is the tertiary referral center for MND in southwest China (38).

As of 2018, there were 1,201 patients with sporadic ALS enrolled in the registry. An additional 59 cases of familial or juvenile ALS are also included in the registry. The age of onset for ALS ranged from 22 to 86 years (mean 54.1, median 55.6 years). ~13% of patients developed symptoms before 40 years of age. The mean diagnostic delay within this population was 17 months. ~78% of patients had spinal onset, 23% had bulbar onset, and 0.3% had respiratory onset. The mean ALSFRS-R score was 37.8. ~16% of patients were former smokers, 18% were current smokers, and 66% were never smokers.

Demographic information, personal history (cigarette smoking, drinking, pesticide exposure, and living environment) is recorded via questionnaires. Disease-related data, including age of onset, the region of symptom onset (upper limb, lower limb, or bulbar), disease duration, diagnostic delay, and family history are also collected at baseline (initial enrollment).

Patients are followed up using telephone or face-to-face interviews at 3 or 6 month intervals by neurologists. The ALSFRS-R score, progression of symptoms, and treatments, as well as newly developed comorbidities and additional medications are recorded at each interview.

In a recent paper, Wei et al. (38) explored the natural history, clinical features, and survival of patients presenting with ALS/MND. The authors assessed 1,201 patients. Cases were classified according to established phenotypes. The investigators reported that 1,157 patients with complete clinical information were evaluable. The mean age of onset, diagnostic delay, smoking status, exposure to pesticides, and mean survival time were significantly different among different phenotypes. The median survival time was approximately 3 years for all patients. The 3 year survival proportion was 62.1%, the 5 year survival proportion was 39.6%, and the 10 year survival proportion was 22.0%. The disease phenotype was an independent predictor of survival (hazard ratio = 0.825, *p* < 0.001), after adjustment for other variables (38).

It should be noted that hospital-based registries represent a unique group of patients and data may not be generalizable to the broader Chinese population.

## Discussion

The overall objective of this report was to identify and describe some of the key ALS registries and key patient data collection and sharing initiatives available now that may be suitable and available for future scientific collaborations.

The scope of the targeted review was North America, Europe, Japan and China and other key regions and countries, as available. While the emphasis of the work was larger-scale collaborative registries, networks, and consortia, country-specific and regional registries and subsets were included if they were judged to be of potential value to support the development and delivery of novel epidemiologic and clinical research.

In all, 22 registries and collaborative efforts were highlighted representing over 30,000 patients with ALS across North and South America, Europe, and Asia. When available, details regarding recruitment, enrollment, and the clinical, biological and patient reported outcomes data collected in the registries were provided. Brief commentaries were provided to address the suitability of the registry for further exploration. The potential for collaboration to support the development of epidemiologic data to inform clinical development programs was also discussed.

In addition to insurance claims and administrative data, electronic medical records, and other sources of real-world data, registry data can provide unique insight into rare diseases such as ALS where the conduct of more traditional types of studies (e.g., RCTs) is often infeasible or unethical. Registry data may help advance our understanding of the causes of disease, natural history, and treatment patterns of disease and may even inform the most appropriate distribution of resources for future research. The registries also provide an opportunity to obtain patient- (and caregiver-) relevant information which is important to assess patient-perceived burden and to address regulatory and Health Technology Assessment (HTA) initiatives regarding patient-centered outcomes (e.g., longer PRO or qualitative measures that are not feasible within a clinical trial due to patient burden).

A number of country and regional efforts have been developed in many parts of the world to develop sustainable efforts at collecting real world evidence to accelerate therapy development in ALS. The unique strengths and limitations of each registry are important to consider in the context of the particular research questions and scientific objectives of the work. A notable limitation in the United States is the lack of standardization across states in ALS status as a reportable condition which may limit potential opportunities from a real-world data collection standpoint. Additionally, a lack of uniform data collection protocols may limit the strength of RWD in rare disease population.

Overall, several “wins” have been reported through use of the RWD sources described here, and this is evident through the multiple publications brought forth from these efforts. When strengths are magnified and limitations are taken into account, registries have the potential to provide valuable observational data to inform ALS clinical development programs by identifying targets and clinical endpoints, and for potential contributions to the evaluation of the safety and effectiveness of new indications for approved products. These data can also contribute to the regulatory review of new drugs and satisfy post-approval requirements. Innovative approaches to data linkage with biospecimen collection and collaboration with other registries can only further advance the utility of this type of data.

An important limitation to this review was its targeted, rather than systematic approach, which may have resulted in a collection of real-world data sources that is not exhaustive of those available in the ALS field. This limitation was mitigated to some extent by seeking recommendations from subject matter experts. However, the current review synthesizes information on some of the most prominent and promising real world evidence sources for ALS research.

## Author Contributions

SC, TR, CS, SH, CC, NZ, VM, and SD all made substantial contributions to the conception of the work. They participated in the drafting, review, and final approval of the work.

## Funding

Funding for this work was provided by Takeda Development Center Americas, Inc.

## Conflict of Interest

SC, TR, and CS are independent contractors with CERobs Consulting, LLC who were contracted through Takeda Pharmaceuticals to conduct this research. SH, CC, NZ, VM, and SD are paid employees of Takeda Pharmaceuticals. This study received funding from Takeda Pharmaceuticals. The funder had the following involvement with the study: study design, decision to publish, and review of the manuscript.

## Publisher's Note

All claims expressed in this article are solely those of the authors and do not necessarily represent those of their affiliated organizations, or those of the publisher, the editors and the reviewers. Any product that may be evaluated in this article, or claim that may be made by its manufacturer, is not guaranteed or endorsed by the publisher.

## Footnotes

^1^ https://www.atsdr.cdc.gov/features/alsregistryanniversary/index.html#:\sim:text=It%20is%20the%20only%20population,U.S.%20are%20living%20with%20ALS

^2^ https://www.cdc.gov/als/

^3^ https://www.cdc.gov/als/ALSNationalBiorepository.html

^4^ https://cndr.org/

^5^ https://www.projectmine.com/

^6^ https://www.projectmine.com/research/data-browser/

^7^ https://www.neuroepidemiologia.edu.uy/web/index.php/lineas-de-investigacion/laenals
